# Curcumin Ameliorates Lead-Induced Hepatotoxicity by Suppressing Oxidative Stress and Inflammation, and Modulating Akt/GSK-3β Signaling Pathway

**DOI:** 10.3390/biom9110703

**Published:** 2019-11-05

**Authors:** Ahlam Alhusaini, Laila Fadda, Iman H. Hasan, Enas Zakaria, Abeer M. Alenazi, Ayman M. Mahmoud

**Affiliations:** 1 Pharmacology and Toxicology Department, Faculty of Pharmacy, King Saud University, Riyadh 11451, Saudi Arabia; lfadda@ksu.edu.sa (L.F.); ihasan@ksu.edu.sa (I.H.H.); malharbi10@yahoo.com (A.M.A.); 2 Pharmaceutics Department, College of Pharmacy, King Saud University, Riyadh 11451, Saudi Arabia; dhanahi@ksu.edu.sa; 3 Physiology Division, Zoology Department, Faculty of Science, Beni-Suef University, Beni-Suef 62514, Egypt

**Keywords:** Lead, Curcumin, GSK-3β, JNK, NF-κB, oxidative stress

## Abstract

Lead (Pb) is a toxic heavy metal pollutant with adverse effects on the liver and other body organs. Curcumin (CUR) is the principal curcuminoid of turmeric and possesses strong antioxidant and anti-inflammatory activities. This study explored the protective effect of CUR on Pb hepatotoxicity with an emphasis on oxidative stress, inflammation and Akt/GSK-3β signaling. Rats received lead acetate and CUR and/or ascorbic acid (AA) for seven days and samples were collected for analyses. Pb(II) induced liver injury manifested by elevated serum alanine aminotransferase (ALT), aspartate aminotransferase (AST) and lactate dehydrogenase (LDH), as well as histopathological alterations, including massive hepatocyte degeneration and increased collagen deposition. Lipid peroxidation, nitric oxide, TNF-α and DNA fragmentation were increased, whereas antioxidant defenses were diminished in the liver of Pb(II)-intoxicated rats. Pb(II) increased hepatic NF-κB and JNK phosphorylation and caspase-3 cleavage, whereas Akt and GSK-3β phosphorylation was decreased. CUR and/or AA ameliorated liver function, prevented tissue injury, and suppressed oxidative stress, DNA damage, NF-κB, JNK and caspase-3. In addition, CUR and/or AA activated Akt and inhibited GSK-3β in Pb(II)-induced rats. In conclusion, CUR prevents Pb(II) hepatotoxicity via attenuation of oxidative injury and inflammation, activation of Akt and inhibition of GSK-3β. However, further studies scrutinizing the exact role of Akt/GSK-3β signaling are recommended.

## 1. Introduction

Lead (Pb) is a non-essential trace element that is widely used in industries, such as battery manufacturing and recycling. It is a toxic heavy metal pollutant and its worldwide emission rate is very high [1,2]. Pb can affect different tissues of the human body and its long-term exposure is linked to neurological disorders, liver injury, cardiovascular disease, osteoporosis and various cancers [3,4,5]. Inhalation, skin absorption, and ingestion of contaminated food or water are the common routes of Pb poisoning. It has been estimated that Pb contamination causes 540,000 deaths annually [6], and 26 million are at the risk of poisoning [7]. Experimental studies have demonstrated that exposure to low-doses of Pb provokes liver injury in rodents [8,9,10]. The ionic properties of Pb, oxidative stress and diminished cellular antioxidants are the main causes underlying Pb toxicity [11]. Pb can replace mono- and divalent cations in enzymes and other proteins, resulting in disrupted cell metabolism, enzyme activities, signaling pathways and ionic transportation [12]. Moreover, Pb promotes oxidative injury via triggering the production of reactive oxygen species (ROS) [13]. Besides oxidative stress, Pb toxicity has been associated with the development of inflammatory responses and cell death [14]. The adverse health effects of Pb have been thought to be mediated by inflammation [14]. Accordingly, male subjects with high blood Pb showed leukocytosis and increased tumor necrosis factor (TNF)-α levels [15]. In Pb-exposed workers, inflammatory mediators were increased and showed positive correlation with blood Pb levels [16].

Glycogen synthase kinase 3 (GSK-3) is a ubiquitously expressed serine/threonine kinase with distinctive functions in different cells. It exists in α and β isoforms which share a homologues kinase domain, but possess different functions [17]. It is downstream of insulin, growth factors, and other major signaling pathways. The activity of GSK3 is primarily controlled by phosphorylation-mediated inactivation; however, sequestration and subcellular localization and other inactivation methods are also known [18]. GSK3β regulates cell proliferation and differentiation, metabolism, apoptosis and other cellular activities in different cells [19,20,21]. In resting cells, GSK-3β is active and stimulation results in its inhibition by Ser9 phosphorylation [17]. Inhibition of GSK3 has been recently shown to accelerate liver regeneration in a mouse model of drug-induced hepatotoxicity [22], and to protect against cell death provoked by ischemia/reperfusion (I/R) [23]. In contrast, increased activity of GSK-3β suppressed autophagy and promoted liver injury in mice [24]. These findings introduce an evidence that inhibition of GSK-3β might protect the liver against toxicity induced by heavy metals.

Curcumin (CUR) is a plant-derived polyphenol with powerful anti-inflammatory and antioxidant activities [25,26]. CUR prevented oxidative damage and apoptosis in a rodent model of gentamicin-induced hepato- and nephrotoxicity [25,26]. Besides its ability to suppress oxidative stress and inflammation, CUR possesses anticancer, anti-atherosclerotic, anti-diabetic and anti-obesity properties [27]. Very recently, CUR prevented oxidative stress and cerebellar toxicity in Pb-induced rats [28]. CUR can affect GSK-3 activity in different diseases (reviewed in [29]). CUR has been reported to fit into the binding pocket of GSK-3 as revealed by molecular docking, and inhibited GSK-3β *in vitro* with IC50 of 66.3 nM [30]. In addition, a recent computational simulation study demonstrated the inhibitory effect of CUR and its conjugates with retinoic acid on GSK-3β [31]. Herein, we investigated the protective effect of CUR on Pb hepatotoxicity. On the basis of previous studies, we hypothesized that CUR can prevent oxidative stress, inflammatory response and apoptosis and inhibit GSK-3β in lead acetate (Pb(Ac)_2_)-induced rats.

## 2. Materials and Methods

### 2.1. Experimental Animals and Treatments

Thirty male Wistar rats weighing 180–190 g were kept for one week before the onset of the experiment. The animals were housed under standard conditions (23 ± 2 °C and 50–60% humidity) and supplied a chow diet and water *ad libitum*. The experimental protocol was approved by the Animal Care and Use Committee of the College of Pharmacy, King Saud University (Ethical approval no.: KSU-SE-19-33).

The animals were allocated into 5 groups (*n* = 6) as follows:

Group I: received vehicles and served as a control.

Group II: received 50 mg/kg Pb(Ac)_2_ [32] intraperitoneally (i.p.) for seven consecutive days.

Group III: received 200 mg/kg ascorbic acid (AA) [33] orally and 50 mg/kg Pb(Ac)_2_ i.p. for seven consecutive days.

Group IV: received 200 mg/kg CUR [28] orally and 50 mg/kg Pb (Ac)_2_ i.p. for seven consecutive days.

Group V: received 200 mg/kg AA and 200 mg/kg CUR orally and 50 mg/kg Pb(Ac)_2_ i.p. for seven consecutive days.

Pb(Ac)_2_ was purchased from Sigma (St. Louis, MO, USA) and dissolved in physiological saline. Rats in Group I received saline i.p. for seven days. AA and CUR (Sigma, St. Louis, MO, USA) were dissolved in 1% carboxymethyl cellulose (CMC). Rats in groups I and II received 1% CMC orally for seven days. At day 8, all rats were sacrificed under anesthesia and blood was collected for serum separation. After dissection, liver was removed, weighed and a 10% w/v homogenate was prepared in cold phosphate buffered saline (PBS). The homogenate was centrifuged, and supernatant was collected for the assessment of lipid peroxidation (LPO), glutathione (GSH), nitric oxide (NO) and superoxide dismutase (SOD). Pieces from the liver were fixed in 10% neutral buffered formalin while others were kept frozen at −80 °C.

### 2.2. Determination of Liver Function Markers

Serum alanine aminotransferase (ALT), aspartate aminotransferase (AST) and lactate dehydrogenase (LDH) were assayed using reagent kits (Randox, Crumlin, UK) following the provided instructions.

### 2.3. Determination of LPO, NO, Antioxidants and TNF-α

LPO was determined in the liver homogenate by assaying malondialdehyde (MDA) as previously described [34]. NO was assayed using Griess reagent [35], and the antioxidants GSH and SOD were determined according to Beutler et al. [36] and Marklund and Marklund [37], respectively. TNF-α was assayed using R&D (Minneapolis, MN, USA) ELISA kit according to the provided instructions.

### 2.4. Histological Examination

The liver samples were fixed in 10% neutral buffered formalin for 24 h, dehydrated and embedded in paraffin wax. Then, 5-μm sections were cut, deparaffinized, rehydrated and stained with hematoxylin and eosin (H&E). Other sections were stained with Masson’s trichrome (MT) and all were examined using a light microscope.

### 2.5. Western Blot

The frozen liver samples were homogenized in RIPA buffer with proteinase and phosphatase inhibitors and protein concentration was determined using Bradford protein assay kit (BioBasic, Markham, Canada). 40 µg proteins were subjected to 10% SDS/PAGE and transferred to nitrocellulose membranes which were blocked using 5% skimmed milk in tris buffered saline/tween 20 (TBST). The membranes were incubated with antibodies against nuclear factor-kappaB (NF-κB) p65, phosphorylated c-Jun N-terminal kinase (pJNK), JNK, cleaved caspase-3, pAkt Ser473, Akt, pGSK-3β Ser9, GSK-3β and β-actin overnight at 4 °C. After washing in TBST, the membranes were probed with the secondary antibodies. All antibodies were supplied by Novus Biologicals (Centennial, CO, USA). The membranes were washed with TBST and developed using enhanced chemiluminescence detection kit (BIO-RAD, Hercules, CA, USA). The blots were scanned, and the band intensity was quantified using ImageJ (version 1.32j, NIH, USA).

### 2.6. Determination of DNA Fragmentation

DNA fragmentation was analyzed by agarose gel electrophoresis. In addition, DNA fragmentation was quantified in the liver of control and treated rats as previously described [38]. This method is based on tissue lysis followed by centrifugation to generate intact chromatin (pellet) and fragmented DNA (supernatant). After protein precipitation, the samples were treated with diphenylamine and absorbance of the developed color was measured at 600 nm. The results were presented as percent of the control.

### 2.7. Statistical Analysis

Statistical analysis was performed using GraphPad Prism (GraphPad Software, La Jolla, CA, USA), and all statistical comparisons were made by one-way analysis of variance (ANOVA) test followed by Tukey’s test post hoc analysis. The differences were considered statistically significant at *p* < 0.05. The results were presented as mean ± SEM (standard error of mean).

## 3. Results

### 3.1. CUR Prevents Pb(II)-Induced Liver Injury

**Figure 1:**
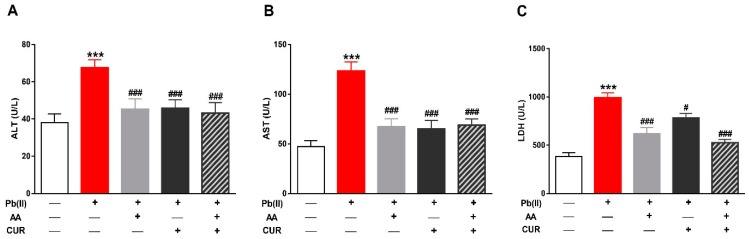
Curcumin (CUR) prevents Pb(II)-induced liver injury in rats. CUR, AA and their combination reduced serum alanine aminotransferase (ALT) (**A**), aspartate aminotransferase (AST) (**B**) and lactate dehydrogenase (LDH) (**C**) in Pb(II)-intoxicated rats. Data are expressed as mean ± SEM, (*n* = 6). *** *p* < 0.001 versus Control. ^#^ *p* < 0.05 and ^###^ *p* < 0.001 versus Pb(II).

Rats received Pb(II) exhibited liver injury manifested by the significantly increased (*p* < 0.001) ALT (Figure 1A), AST (Figure 1B) and LDH (Figure 1B) in serum. Concurrent administration of AA ameliorated all liver function markers significantly (*p* < 0.00) in Pb(II)-induced rats. Similarly, CUR ameliorated ALT (*p* < 0.001), AST (*p* < 0.001) and LDH (*p* < 0.05). Pb(AC)_2_-induced rats received both AA and CUR and showed significantly (*p* < 0.001) alleviated liver function markers.

**Figure 2:**
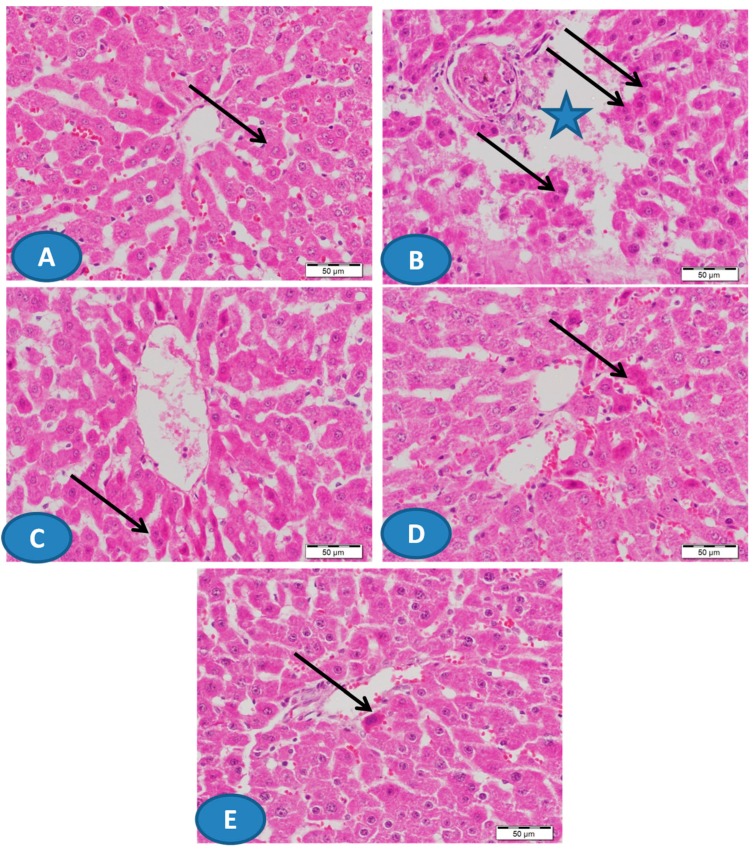
Photomicrographs of hematoxylin and eosin (H&E)-stained sections from liver of (**A**) control rats showing normal hepatic architecture with normal hepatocytes (arrow) and blood sinusoids, (**B**) Pb(II)-intoxicated rats showing focal areas of massive hepatic degeneration (star) and many degenerated hepatocytes (arrows), and (**C**–**E**) Pb(II)-administered rats treated with CUR (**C**) and AA (**D**) showing improvement of the hepatic architecture with degenerated hepatocytes (arrows), and CUR/AA (**E**) showing apparently normal hepatic architecture with very few degenerated hepatocytes (arrow). (X400, Scale bare 50 µm).

The hepatoprotective effect of CUR and/or AA was supported by the histological examination of H&E-stained sections (Figure 2). While the control rats showed normal liver structure (Figure 2A), the administration of Pb(II) caused massive hepatic degeneration along with other manifestations (Figure 2B). Treatment of the rats with CUR and/or AA markedly prevented the histological alterations induced by Pb(II) as represented in Figure 2C–E.

**Figure 3:**
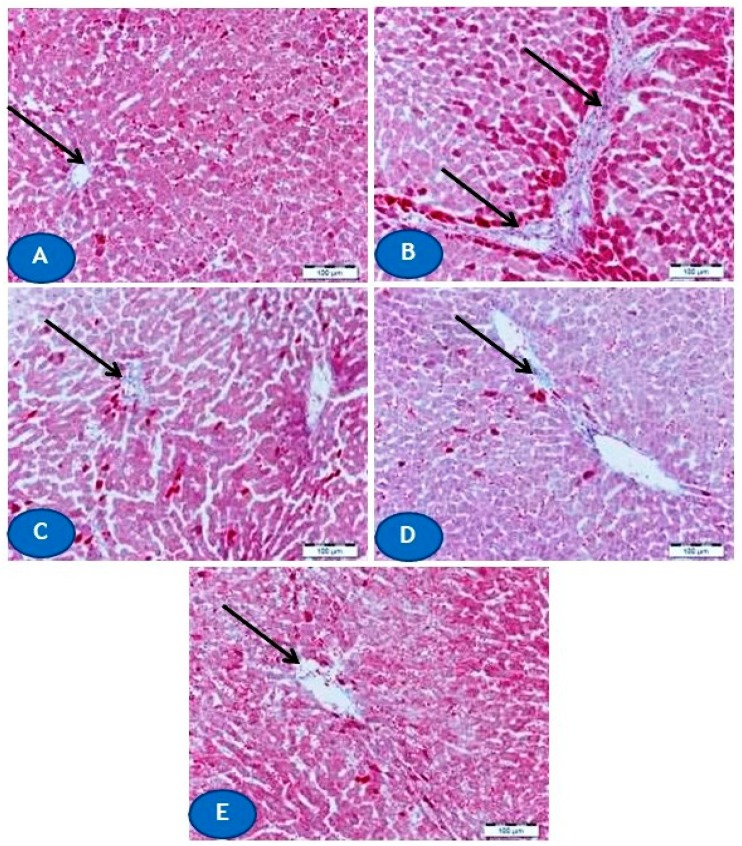
Photomicrographs of Masson’s trichrome (MT)-stained sections from liver of (**A**) control rats showing normal amount and distribution of collagen fibers around blood vessels (arrow), (**B**) Pb(II)-intoxicated rats showing an increase in collagen deposition (arrows), and (**C**–**E**) Pb(II)-administered rats treated with CUR (**C**) and AA (**D**) showing marked decrease in collagen deposition (arrow), and CUR/AA (**E**) showing apparently normal distribution of collagen fibers (arrow). (X400, Scale bare 50 µm).

Furthermore, microscopic examination of MT-stained liver sections revealed a remarkable increase in collagen deposition in Pb(II)-intoxicated rats (Figure 3B) when compared with the normal distribution and amount of collagen in normal rats (Figure 3A). Concomitant treatment with CUR and/or AA prevented the increase in collagen deposition promoted by Pb(II) as depicted in Figure 3C–E.

### 3.2. CUR Attenuates Oxidative Stress in Pb(II)-Induced Rats

**Figure 4:**
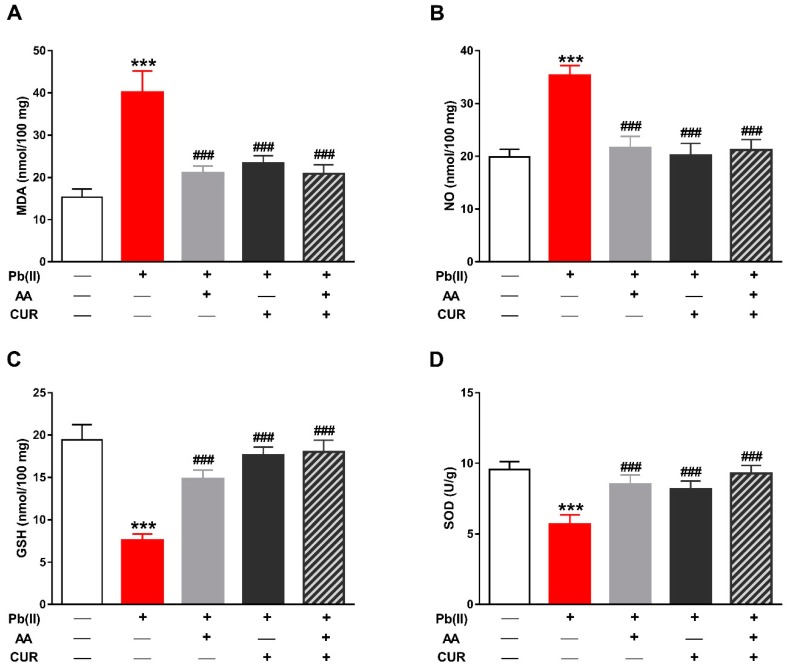
CUR attenuates oxidative stress in Pb(II)-induced rats. CUR, AA and their combination reduced malondialdehyde (MDA) (**A**) and nitric oxide (NO) (**B**), and increased glutathione (GSH) (**C**) and superoxide dismutase (SOD) activity (**D**) in liver of Pb(II)-intoxicated rats. Data are expressed as mean ± SEM, (*n* = 6). *** *p* < 0.001 versus Control and ^###^ *p* < 0.001 versus Pb(II).

The role of oxidative stress in Pb(II) hepatotoxicity and the protective role of CUR and/or AA was assessed through the determination of LPO, NO and the antioxidants GSH and SOD. Pb(II) increased the LPO marker MDA in the liver of rats when compared with the control group (*p* < 0.001; Figure 4A). In contrast, Pb(II)-intoxicated rats treated with CUR and/or AA showed significantly decreased hepatic MDA levels (*p* < 0.001). Similarly, hepatic NO was markedly elevated in Pb(II)-intoxicated rats (*p* < 0.001; Figure 4B). Concomitant administration of CUR and/or AA reduced hepatic NO levels significantly (*p* < 0.001) when compared with the Pb(II)-induced group.

In comparison with the control group, GSH content (Figure 4C) and SOD activity (Figure 4D) were significantly declined in Pb(II)-induced rats (*p* < 0.001). In contrast, rats received CUR and/or AA concomitantly with Pb(II) exhibited noticeably improved hepatic GSH and SOD.

### 3.3. CUR Inhibits Inflammation in Pb(II)-Induced Rats

**Figure 5:**
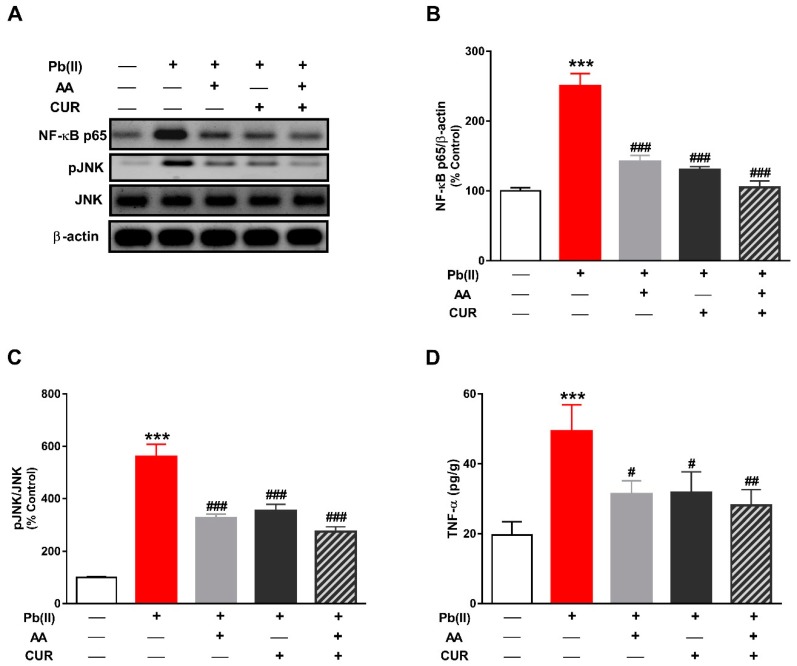
CUR inhibits inflammation in Pb(II)-induced rats. (**A**) Representative blots of NF-κB p65, pJNK, JNK and β-actin. (**B**–**D**) CUR, AA and their combination decreased the phosphorylation levels of NF-κB (**B**) and JNK (**C**), and TNF-α levels (**D**) in liver of Pb(II)-intoxicated rats. Data are expressed as Mean ± SEM, (*n* = 6). *** *p* < 0.001 versus Control. ^#^ *p* < 0.05, ^##^ *p* < 0.01 and ^###^ *p* < 0.001 versus Pb(II).

NF-κB is a redox-sensitive transcription factor that elicits the release of pro-inflammatory cytokines in response to excess ROS. Pb(II)-induced rats exhibited a significant increase in the phosphorylation levels of hepatic NF-κB (*p* < 0.001; Figure 5A,B). In addition, Pb(II) provoked a significant increase in JNK phosphorylation in the liver of rats (*p* < 0.001; Figure 5A,C). The inflammatory response in Pb(II)-induced rats was confirmed by the significant increase in hepatic TNF-α (*p* < 0.001; Figure 5D). Treatment with CUR and/or AA significantly reduced the phosphorylation levels of NF-κB and JNK, and TNF-α in the liver of Pb(II)-intoxicated rats.

### 3.4. CUR Attenuates Apoptosis in Pb(II)-Induced Rats

**Figure 6:**
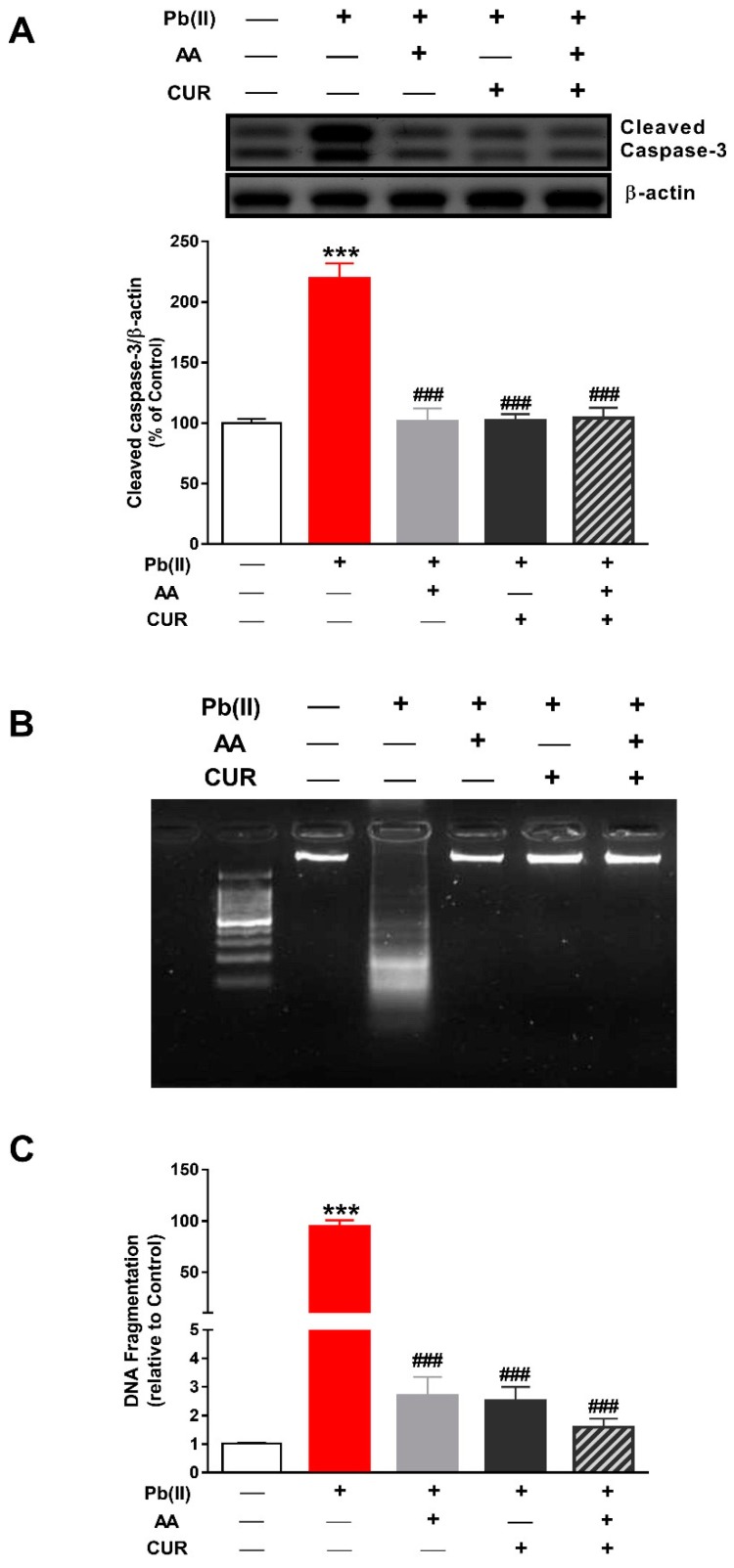
CUR attenuates apoptosis in Pb(II)-induced rats. CUR, AA and their combination diminished caspase-3 activation (**A**) and DNA fragmentation (**B**,**C**) in liver of Pb(II)-intoxicated rats. (**B**,**C**) Data are expressed as mean ± SEM, (*n* = 6). *** *p* < 0.001 versus control and ^###^ *p* < 0.001 versus Pb(II).

Cleaved caspase-3 and DNA fragmentation were determined to assess the antiapoptotic efficacy of CUR and/or AA in Pb(II) -intoxicated rats. Caspase-3 was significantly activated in the liver of rats received Pb(II) as shown in Figure 6A. Concomitant administration of CUR and/or AA diminished the activation of caspase-3 significantly (*p* < 0.001) in Pb(II)-intoxicated rats.

Analysis of DNA fragmentation using agarose gel electrophoresis revealed marked DNA fragmentation in the liver of Pb(II)-intoxicated rats (Figure 6B). This result was confirmed by the colorimetric assay which showed a significant DNA fragmentation in the liver of rats received Pb(II) (Figure 6C). CUR, AA and their combination inhibited Pb(II)-induced DNA fragmentation in the liver of rats.

### 3.5. CUR Up-Regulates Akt/GSK-3β Signaling in Pb(II)-Induced Rats

**Figure 7:**
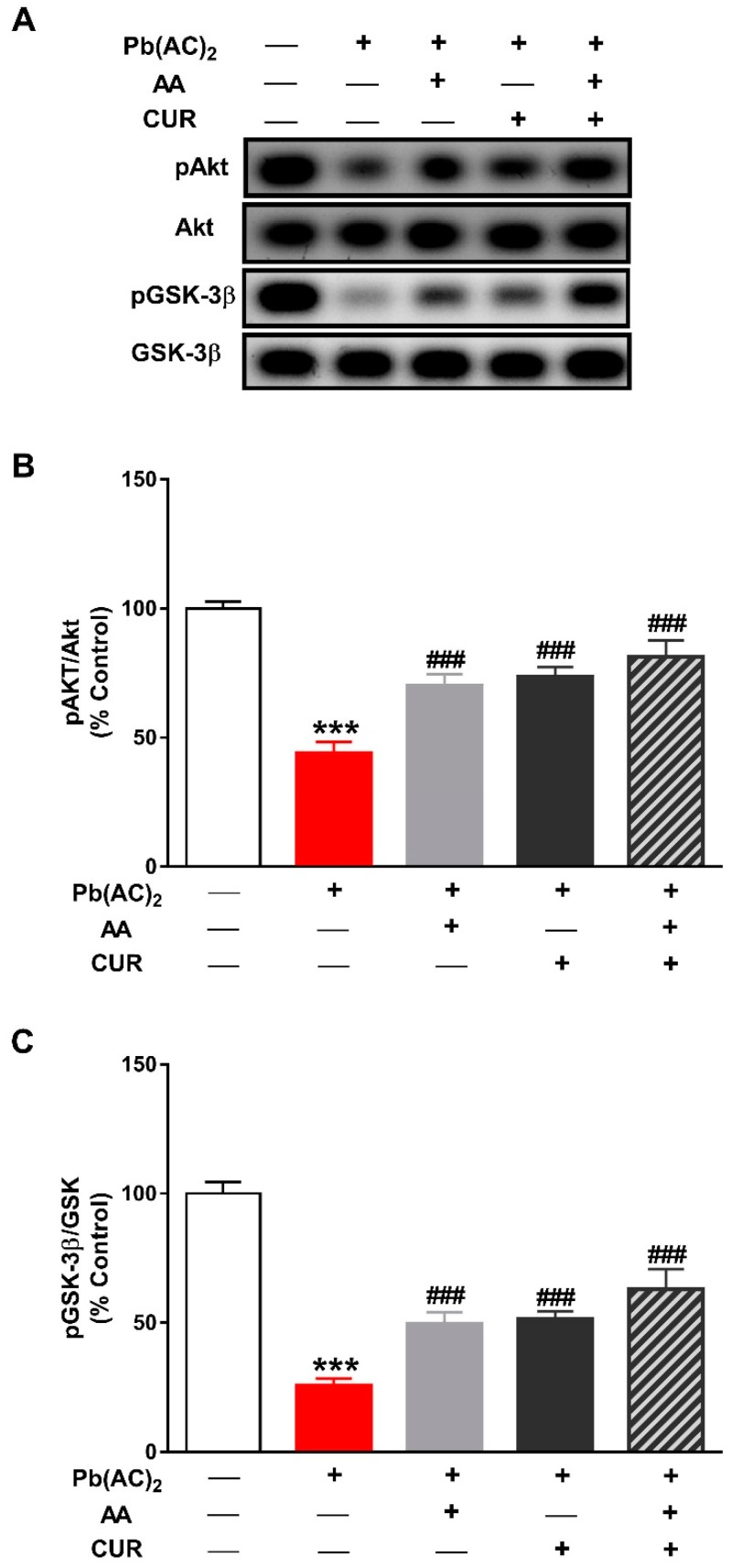
CUR up-regulates Akt/GSK-3β signaling in in Pb(II)-induced rats. (**A**) Representative blots of pAkt, Akt, pGSK-3β and GSK-3β. (**B**,**C**) CUR, AA and their combination increased the phosphorylation levels of Akt (**B**) and GSK-3β (**C**) in liver of Pb(II)-intoxicated rats. Data are expressed as Mean ± SEM, (*n* = 6). *** *p* < 0.001 versus control and ^###^ *p* < 0.001 versus Pb(II).

The phosphorylation levels of Akt were significantly reduced in the liver of Pb(II)-intoxicated rats (*p* < 0.001) as depicted in Figure 7A,B. GSK-3β phosphorylation was also diminished in rats received Pb(II) when compared with the control group (Figure 7A,C). Concurrent administration of CUR and/or AA significantly increased the levels of pAkt and pGSK-3β in the liver of Pb(II)-intoxicated rats (*p* < 0.001).

## 4. Discussion

The toxic effect of Pb on different body organs particularly the liver and kidney has been well-documented. Studies have demonstrated that liver and kidney are the most common depository of Pb [39]. CUR has shown promising protective effects against drug-induced hepato- and nephrotoxicity [25,26]. In Pb-induced animals, CUR ameliorated hepatotoxicity [32] and cerebellar toxicity [28]. However, the mechanisms underlying the hepatoprotective efficacy of CUR are not fully understood. Herein, we investigated the effect of CUR and/or AA on oxidative stress, inflammation and Akt/GSK-3β signaling in Pb(II)-intoxicated rats.

Rats received Pb(II) exhibited liver dysfunction and injury evidenced by the elevated serum levels of ALT, AST and LDH along with the histopathological alterations. Accordingly, AST and ALT were elevated in serum of rats received Pb(Ac)_2_ at dose levels of 50 mg/kg for seven days [32] or 0.4% in drinking water for eight weeks [40]. In both studies, Pb(Ac)_2_ induced tissue injury manifested by the fibrous tissue proliferation and congestion of portal blood vessels [32,40]. Our study added support to these findings where the histopathological examination revealed focal areas of massive hepatic degeneration and increased deposition of collagen in liver of Pb(II)-intoxicated rats. Concurrent administration of CUR, alone or combined with AA, for seven days prevented Pb(II)-induced tissue injury and collagen deposition, and ameliorated the circulating levels of liver function markers. Similar to our findings, rats received 200 mg/kg CUR for four weeks and challenged with Pb(AC)_2_ during the 4th week showed significant decrease in serum ALT and AST [32].

The hepatoprotective efficacy of CUR could be directly attributed to its chelating and antioxidant properties. Given the ionic properties of Pb and its ability to replace mono- and divalent cations in enzymes [12], it is noteworthy to assume that CUR chelated Pb(II) and prevented its deposition in the liver of rats. This notion is supported by a previous study showing that four-week CUR administration reduced serum Pb levels in Pb(Ac)_2_-induced rats [32]. In addition, CUR reduced Pb accumulation in the cerebellum of rats received 50 mg/kg Pb(Ac)_2_ for four weeks as recently described [28]. Besides its ion chelating properties, CUR prevented oxidative stress in liver of Pb(II)-intoxicated rats. Our results showed a significant increase in hepatic LPO and NO, and diminished GSH and SOD activity in Pb(II)-intoxicated rats, indicating oxidative stress. Increased ROS generation and declined antioxidant defenses represent a main culprit behind Pb toxicity [11,13]. Pb promote oxidative injury via triggering the production of ROS [13]. ROS provoke injury through oxidizing lipids and proteins, inactivating antioxidant enzymes and triggering DNA damage [41]. Previous studies have demonstrated altered expression of antioxidant enzymes in liver [32] and increased LPO and diminished SOD in cerebellum [28] of Pb(Ac)_2_-induced rats. Here, CUR reduced LPO and NO, and boosted cellular antioxidants in liver of Pb(II)-intoxicated rats, demonstrating a potent antioxidant efficacy. In accordance, CUR increased the gene expression of hepatic antioxidant enzymes [32] and reduced cerebellar LPO [28] in Pb(Ac)_2_-induced rats. Besides its radical-scavenging activity, CUR has been demonstrated to modulate the nuclear-factor erythroid 2-related factor 2 (Nrf2) in different models of liver injury [42,43,44]. Nrf2 is a redox-sensitive transcription factor that regulates the transcription of antioxidant and cytoprotective genes and protects against oxidative stress [45]. Accordingly, activation of Nrf2 signaling has been reported to protect against hepatotoxicity induced by different agents, including methotrexate, cyclophosphamide and diethylnitrosamine [46,47,48,49]. In murine models of carbon tetrachloride [42] and lipopolysaccharide/d-galactosamine-induced liver injury [44], and hepatic steatosis [50], CUR supplementation activated Nrf2 signaling and exerted hepatoprotective effects. Recently, *in silico* analysis revealed that CUR interacts directly with Nrf2 [51]. Therefore, attenuation of oxidative stress represents a main part of the hepatoprotective mechanism of CUR against Pb toxicity. This notion is supported by the ameliorated liver function and antioxidant defenses, and diminished LPO and NO in Pb(II)-intoxicated rats treated with AA.

Besides oxidative stress, inflammation has also been implicated in the adverse toxic effects of Pb [14]. In the present study, Pb induced an inflammatory response as shown by increased phosphorylation of NF-κB and TNF-α levels. NF-κB is a transcription factor that is activated by ROS to induce the production of inflammatory mediators, including TNF-α, interleukin (IL)-6, inducible NO synthase (iNOS) and others. TNF-α is released by activated macrophages and participates in both local and systemic inflammation [52]. The inflammatory response elicited by Pb has been reported in male subjects with high blood Pb who showed leukocytosis and increased TNF-α [15], and in Pb-exposed workers where inflammatory mediators were increased with increased blood Pb levels [16]. Healthy blood mononuclear cells (PBMCs) treated with 1 ng/mL LPS and different concentrations of lead chloride showed an increase in TNF-α expression [53]. In addition, increased expression of TNF-α and transforming growth factor (TGF)-β1 has been demonstrated in liver of rats exposed to Pb(Ac)_2_ for seven days [32]. This can explain the increased collagen deposition in the liver of Pb(Ac)_2_-intoxicated rats in the current study. TGF-β is a core pathway of fibrosis [54], and recent work from Mahmoud’s laboratory demonstrated that the activation of TGF-β1/Smad3 signaling was associated with inflammation and fibrosis in the liver of rats [47,55].

Moreover, Pb(II)-intoxicated rats exhibited an increase in JNK phosphorylation. JNK is a MAPK family member that catalyzes the phosphorylation of several proteins within the liver [56]. JNK is activated by diverse stimuli, including TNF-α and ROS [57]. Sustained ROS accumulation and activation of JNK are associated with apoptosis [58]. TNF-α provokes recruitment of pJNK and Bax to the outer mitochondrial membrane, leading to generation of ROS and sustained activation of JNK [59]. Upon TNF-α stimulation, caspase-8 cleaves Bid in a JNK-dependent manner and cleaved Bid induces the release of mitochondrial cytochrome *c* and subsequent activation of caspase-3, resulting in cell death [60]. Therefore, TNF-α-mediated recruitment of pJNK to the mitochondria is an important step in hepatocyte death [59]. Herein, the liver of Pb(II)-intoxicated rats exhibited significant activation of caspase-3, demonstrating apoptotic cell death. Pb-induced apoptosis in the liver of rats was further supported by the observed DNA damage.

Interestingly, CUR alone and combined with AA suppressed inflammation, DNA damage and apoptosis in liver of rats received Pb(II). CUR diminished NF-κB and JNK phosphorylation, caspase-3 activation and DNA damage, demonstrating potent anti-inflammatory and anti-apoptotic activities. Given that AA attenuated inflammation in Pb(II)-intoxicated rats, it could be assumed that the suppression of ROS-mediated NF-κB activation plays a major role in the anti-inflammatory activity of CUR. In addition, CUR inhibits the inhibitory factor I-kappa B kinase activity and hence block pro-inflammatory gene expression elicited by NF-κB [61]. CUR decreased the gene expression of TGF-β1 and monocyte chemoattractant protein (MCP)-1 when administered before and during Pb(Ac)_2_ administration in rats [32]. In an experimental model of I/R injury, CUR exerted an anti-inflammatory effect mediated via NF-κB inhibition [62]. Moreover, CUR inhibited NF-κB and JNK activation in bisphenol A-induced hepatocytes *in vitro* [63]. In the same experiment, the JNK inhibitor SP600125 exerted beneficial effects [63], pointing to the role of JNK inhibition in the anti-inflammatory activity of CUR. Therefore, CUR possesses a dual antioxidant and anti-inflammatory activity that mediated its protective and anti-apoptotic effects against Pb hepatotoxicity.

To further explore the hepatoprotective mechanism of CUR, we determined its effect on Akt/GSK-3β. Our results showed decreased Akt Ser473 and GSK-3β Ser9 phosphorylation in liver of Pb(II)-intoxicated rats. Given that GSK-3β is active in resting cells and its activity is controlled by phosphorylation-mediated inactivation [17], these data show that GSK-3β is active in liver of Pb(II)-intoxicated rats secondary to the suppressed Akt. Activation of GSK-3β has been reported in animal models of drug hepatotoxicity and I/R and its inhibition accelerated liver regeneration and prevented cell death [22,23,24]. The role of GSK-3β in cell death is supported by the fact that PI3K/Akt signaling both suppress cell death and inhibits GSK-3β [64]. Overexpression of GSK-3β resulted in apoptosis of fibroblasts and neuronal PC12 cells, and expression of a dominant-negative GSK-3β-K85R mutant prevented cell death induced by PI3K inhibition [65]. The pro-apoptotic role of GSK-3 has been suggested to be connected to Bax. This notion was supported by the study of Linseman et al. [66] which suggested that GSK-3 activates Bax by direct phosphorylation and the mitochondrial translocation of Bax has been abolished by mutation of its GSK-3 phosphorylation site. Furthermore, GSK-3 has also been suggested to work in concert with JNK during apoptosis of neuronal cells [67]. Here, CUR increased the phosphorylation of both Akt and GSK-3β in liver of Pb(II)-intoxicated rats. Although the inhibitory effect of CUR on GSK-3β has been shown by computational and experimental approaches [29,30,31], our study introduced new information that modulation of Akt/GSK-3β signaling plays a role in the protective mechanism of CUR against Pb hepatotoxicity. Molecular docking studies showed the fit of CUR into the binding pocket of GSK-3 [30], and computational approaches reveled the inhibitory effect of CUR and its conjugates with retinoic acid on GSK-3β [31]. Using an *in vitro* inhibition assay, CUR inhibited GSK-3β with IC50 of 66.3 nM [30]. In a rat model of diabetic cardiomyopathy (DCM), CUR attenuated oxidative stress, fibrosis and apoptosis. This study suggested that Akt/GSK-3β signaling mediated the beneficial effects of CUR [68].

## 5. Conclusions

**Figure 8:**
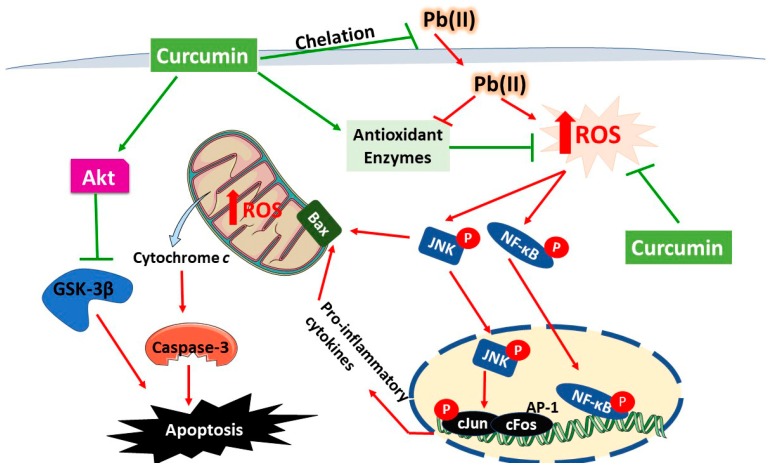
A schematic diagram illustrating the protective mechanism of curcumin against Pb(II) hepatotoxicity. Pb(II) increases ROS generation and activates NF-κB, JNK and GSK-3β, resulting in inflammation and cell death via apoptosis. Curcumin suppresses ROS production, chelates Pb(II), boosts antioxidant defenses and activates Akt signaling. Akt deactivates GSK-3β through phosphorylation at Ser9.

This study demonstrates that CUR prevents Pb hepatotoxicity by attenuating oxidative stress, inflammation, DNA damage and cell death. Our results introduced new information that Akt/GSK-3β signaling is implicated in Pb-induced liver injury and dysfunction. Accordingly, the protective effect of CUR has been mediated, at least in part, via modulating Akt/GSK-3β signaling in liver of rats (Summarized mechanistic pathways is presented in Figure 8). Therefore, CUR could be employed as a protective agent against Pb toxicity particularly in workers exposed to high levels of Pb. However, further studies using genetic and pharmacological tools are recommended to scrutinize the role of Akt/GSK-3β signaling modulation in the protective potential of CUR against Pb toxicity.
